# Maling bamboo (*Yushania maling)* overdominance alters forest structure and composition in Khangchendzonga landscape, Eastern Himalaya

**DOI:** 10.1038/s41598-022-08483-8

**Published:** 2022-03-16

**Authors:** Kailash S. Gaira, Aseesh Pandey, Sheila Sinha, Hemant K. Badola, Jhony Lepcha, Pitamber P. Dhyani, Nakul Chettri

**Affiliations:** 1 G.B. Pant National Institute of Himalayan Environment (NIHE), Sikkim Regional Centre, PangthangGangtok, Sikkim 737 101 India; 2 G.B. Pant National Institute of Himalayan Environment (NIHE), Kosi-Katarmal, Almora, Uttarakhand 263643 India; 3 International Centre for Integrated Mountain Development (ICIMOD), Kathmandu, Nepal; 4 Present Address: D-408, Aditya Doonshire Apartments, Sailok Phase II, GMS Road, Dehradun, Uttarakhand 248 001 India

**Keywords:** Ecology, Environmental sciences, Natural hazards

## Abstract

The Khangchendzonga Landscape (KL), a part of ‘Himalayan Biodiversity Hotspot’, is known for its unique biodiversity assemblage. In recent years, the KL is experiencing threats to biodiversity due to the biological overdominance of native Maling bamboo (*Yushania maling*). In the present study, we investigated the impacts of the overdominance of *Y. maling* on the forest composition of Singalila National Park (SNP), Eastern Himalaya, India. Elevational habitats 2400 to 3400 m asl were sampled by laying 69 (10 m × 10 m) forest plots including 51 bamboo plots and 18 non-bamboo plots. Bamboo plots showed significantly (p < 0.05) low species richness and density in both shrub and herb layers which further manifested the low seedling density. Generalized Additive Model (GAM) estimated a significant (p < 0.0001) decline in species richness and density with increasing bamboo density in SNP. Our study projects the overdominance of *Y. maling* has a significant negative impact on forest structure and composition. Therefore, management of invasiveness of *Y. maling* is essential through its optimized removal from the protected areas and utilization in making handicrafts, paper industries etc. to create ecological and economic benefits. Further long-term studies assessing the impacts of *Y. maling* overdominance on forest ecosystems and soil dynamics are recommended.

## Introduction

Biodiversity provides numerous essential services to society; the survival and socio-economic activities of people largely depend upon various natural resources. Forests, hitherto, are considered to provide renewable resources and ecosystem services that support life on the planet^1–3^. However, invasion of invasive alien species^4–6^ and over expansion/dominance of native plant species^7–9^ alter the composition and structures of natural forests, which mostly manifested by the loss of biodiversity at the global scale^10^. Besides, an overabundance of native herbaceous species can appear as dominant element in the forest ecosystem^11,12^. That may disturb the composition and dynamics of the forest ecosystem adversely. Studies revealed that the expansion of bamboo has altered the overall forest composition and dynamics of many ecosystems^6,13–15^.

Bamboo, the fastest growing plant also referred as ‘green gold’, offers incredible economic, cultural and ecological benefits. Owing to its cheap and plentiful availability, bamboo is also known as ‘poor man’s timber’^16^ and especially used as a substitute of the wood^17^ and as food product in numerous cases for human^18–20^ and wildlife^21,22^. Communities extensively use bamboos, for various household purposes such as supporting poles, flooring, frames, partitions, ceiling, walls, thatching, tying, roofing, making doors and windows frames, etc.^23,24^. Various traditional items and modern decorative are crafted from bamboos. Worldwide, they represented by about 1400 species and 107 genera, consisted 79 genera representing 1200 species of woody bamboo, and 28 genera representing 180 species of herbaceous bamboo^6^. India stands second largest diversity center for bamboos after China^25^ and possess 136 species of bamboo belonging to 18 genera and encompasses over 13.96 million ha occupying 17.3% of the total forest and tree cover of the country^26^ (National Bamboo mission, 2022). Over 84 bamboo species are found in Northeastern Himalaya (India) alone and it is considered one of the major bamboo bio-diversity hotspots in the world^19,27,28^. Bamboo inhabits a wide range of ecosystems along sea level to 4000 m asl. and often questioned for its contribution in population dynamics in plant communities^15^. The uncontrolled expansion/ invasion/overdominance of bamboo in Eastern Asia has led to the formation of ‘bamboo forests’^29,30^ that can remodel the entire structure and composition of the native forests. Bamboo species, especially woody bamboos, are known to change native forest structure by rapid invasion through their fast growing nature and clonal reproduction ability^31,32^. After establishing themselves in the forest areas they start competing for space and nutrition and form dense bamboo stands^33^, which ultimately alters dynamics of forest ecosystems^34^. Furthermore, bamboos are reported to influence the early stage forest regeneration patterns by affecting seed dispersal paterns^35^. Similarly, in parts of India, bamboo overdominance is a concern. A native reed bamboo (*Ochlandra travancorica*) has threatened 20.8% of evergreen forests in Western Ghats, similarly *Yushania maling*, a native bamboo species has become alarmingly invasive in Darjeeling Himalaya^36^.

*Yushania maling* (Gamble) R.B. Majumdar & Karthik. previously known as *Arundinaria maling* Gamble (Family: Poaceae; Sub family: Bambusoideae), is a native bamboo species of temperate zones in eastern Himalaya, having a wide elevation range c.a.1800 to 3600 m^36,37^. Recent MaxEnt, GARP based remote sensing studies in parts of Eastern Himalaya, India indicated its gregarious colonization in the temperate coniferous and temperate broadleaf forests^36^.This plant generally grows in clumps (sympodial growth habit) and attains a height upto 2.5–3 m. However, unlike regular clumping bamboos, it develops wider spreading rhizomes, which makes it an invasive semi-running bamboo, which ultimately expected to exclude native species by outgrowing them^36,38^. Due to its invasive nature *Y. maling* has become a threat to protect areas where native species are conserved.

Like other grass, bamboo flowers are borne on compound inflorescences, however, possess unique flowering behavior. Based on flowering behavior, bamboo species can be classified into three groups (i) continuously flowering or annual flowering species; these are mostly herbaceous species with few exceptional woody species such as *Schizostachyum* sp. etc. This type of flowering may occur in different individual bush (clump) of a forest over different time periods year after year, (ii) sporadic species are those species in which, flowering occur only on individual stems (culms) of the same bush (clump) and do not exhibit a uniform flowering pattern in the forest, (iii) gregariously flowering species are mostly woody bamboo species and all plants of a particular species flower at the same time, regardless of differences in geographic locations or climate conditions. In gregarious flowering plants die after flowering. However, in continuous and sporadic flowering, the plants very rarely die. Most woody bamboos including *Y. maling* are thus considered semelparaous (species that reproduce only once in a lifetime) having flowering cycle of > 50 years^32^. This unpredictability and diversity associated with bamboo flowering and their ecological impacts on neighbouring plant communities have led to many fundamental, botanical and ecological researches^27–37^. However, more strong evidences based on specific flowering time studies are required to suggest effective management planning of over-dominating bamboo forests.

Keeping above in view, we conducted the study in bamboo rich protected area, Singalila National Park (SNP), located in Khangchendzonga Landscape (KL), Indian part of Eeastern Himalaya. We studied, whether invasiveness of *Y. maling* has any significant effect on the forest composition in SNP? Therefore, our study in SNP was focused to: (i) compare species (trees, shrubs and herbs) composition in bamboo and non-bamboo plots, and (ii) estimate the changes in forest composition (species richness and density) due to overdominance of native bamboo species *Y. maling*.

## Results

Along the study transect in Singalila National Park, Khangcendzonga Landscape, India, we sampled 36 tree species belonging to 25 genera and 19 families, 25 species of shrub representing 21 genera and 15 families and in herb layer 46 species belonging to 40 genera and 25 families (Supplementary Table 1). *Rhododendron* and *Quercus* were the most dominant genera in tree layer, *Gaultheria* and *Rubus* in shrub layer, and *Anaphalis* and *Carex* in herb layers respectively. *Ericaceae* was the most dominant family in tree and shrub layers while compositae dominated the herb layer (Supplementary Table 1). The GAM estimated a significant (p < 0.001) increase in species richness for shrubs (β = 0.0013; SE = 0.0006), herbs (β = 0.0017; SE = 0.0008) and tree seedlings (β = 0.0066; SE = 0.0005) along elevation, whereas a significant (p < 0.001) decrease indicated for tree saplings (β = − 0.0017; SE = 0.001). However, tree richness was seemingly unaffected along elevation (Table 1). A significant (p < 0.001) increase in species density for trees (β = 0.005; SE = 0.001), shrubs (β = 0.011; SE = 0.002), herbs (β = 0.650; SE = 0.017), and tree seedlings (β = 0.195; SE = 0.006) was estimated along the elevation. However, a significant decline (p < 0.001) was estimated in species density for tree saplings (β = − 0.019; SE = 0.002) and bamboo (*Y. maling*) culms (β = − 0.33; SE = 0.003) along the elevation.

**Table 1:** Species richness in different vegetation layers along the elevation gradient in Singalila National Park of Khagchendzonga Landscape-India.

Parameters	GAM coefficient (β)	SE (β)	P_value_
**Species richness**			
Trees	0.042	0.0002	1.00
Shrubs	0.0013	0.0006	0.001
Herbs	0.0017	0.0008	0.001
Saplings	− 0.0017	0.001	0.001
Seedlings	0.0006	0.0005	0.0001
**Density**			
Tree	0.005	0.001	0.001
Shrubs	0.011	0.002	0.001
Herbs	0.650	0.017	0.001
Saplings	− 0.019	0.002	0.001
Seedlings	0.195	0.006	0.001
Bamboo (*Y. maling*) culms	− 0.330	0.003	0.001

*β* generalized additive model coefficient, *SE* standard error.

We compared the species composition, *i.e.* species richness and density between bamboo and non-bamboo plots (Table 2). Our results showed significantly (p < 0.0001) low species richness of shrubs (1.39 ± 0.14) and herbs (3.80 ± 0.39) in the bamboo plots as compared to non-bamboo plots (4.22 ± 0.48 shrubs and 6.44 ± 0.67 herbs) respectively. Similarly, we observed significant (p < 0.01) low average density of shrubs (14.27 ± 2.60 individual) and herbs (1710.78 ± 328.4825 individual) in bamboo plots as compared to non-bamboo plots (79.11 ± 22.82 shrubs and 3936.11 ± 912.77 herbs) respectively. We found significantly lower (p < 0.0001) tree seedlings’ richness (1.00 ± 0.16) in bamboo plots than those in non-bamboo plots (2.67 ± 0.21) (Table 3). We also observed lower tree seedlings’ density (130.39 ± 24.70 individual/100 m^2^) in bamboo plots than non-bamboo plots (444.44 ± 41.60 individual/100 m^2^).

**Table 2:** Species richness and density of trees, shrubs and herbs between bamboo and non-bamboo plots.

Parameters	Mean ± SE		t_value_	P_value_
	Bamboo plots (Bamboo density = 300.08 ± 32.69; n = 51)	Non-Bamboo plots (Bamboo density = 0; n = 18)		
**Species richness (100 m**^2^**)**				
Trees	2.61 ± 0.18	2.28 ± 0.39	0.779	0.444
Shrubs	1.39 ± 0.14	4.22 ± 0.48	5.565	0.0001
Herbs	3.80 ± 0.39	6.44 ± 0.67	3.398	0.002
**Density (100 m**^2^**)**				
Trees	5.75 ± 0.65	9.11 ± 1.95	1.642	0.115
Shrubs	14.27 ± 2.60	79.11 ± 22.82	2.832	0.012
Herbs	1710.78 ± 328.48	3936.11 ± 912.77	2.794	0.032

Mean values ± SE of the vegetation parameters calculated and compared between bamboo and non-bamboo plots using t-test for independence samples assuming unequal variance.

**Table 3:** Comparison of species richness and density of saplings and seedlings of trees between bamboo and non-bamboo plots.

Parameters	Mean ± SE		t_value_	P_value_
	Bamboo plots (Bamboo density = 300.08 ± 32.69; n = 51)	Non-bamboo plots (Bamboo density = 0; n = 18)		
**Species richness (100 m**^**2**^**)**				
Saplings	1.90 ± 0.21	1.94 ± 0.27	0.122	0.903
Seedlings	1.00 ± 0.16	2.67 ± 0.21	6.227	0.0001
**Density (100 m**^**2**^**)**				
Saplings	20.94 ± 4.40	22.67 ± 5.65	0.241	0.811
Seedlings	130.39 ± 24.70	444.44 ± 41.60	6.492	0.0001

Mean values ± SE of the vegetation parameters calculated and compared between bamboo and non-bamboo plots using t-test for independence samples.

**Figure 1:**
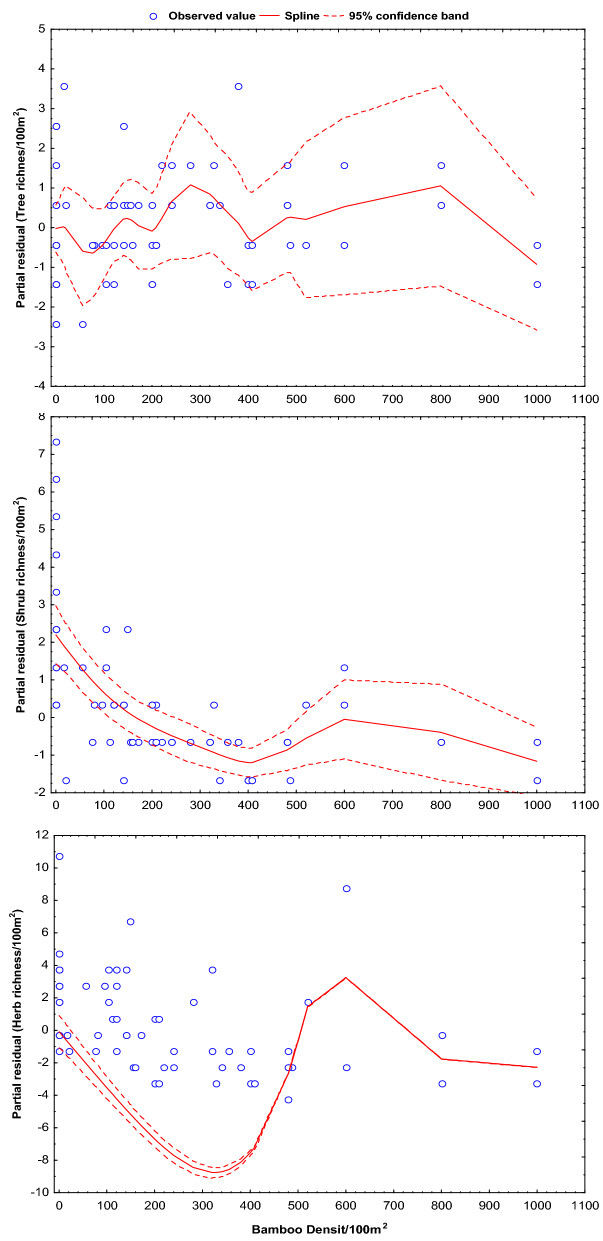
Predicted changes in species richness of trees, shrubs and herbs with respect to increasing density of bamboo. The significance of the smooth best fit model generalized the changing slope as GAM coefficient (β), standard error (SE) and coefficient of determinant (R^2^).

**Figure 2:**
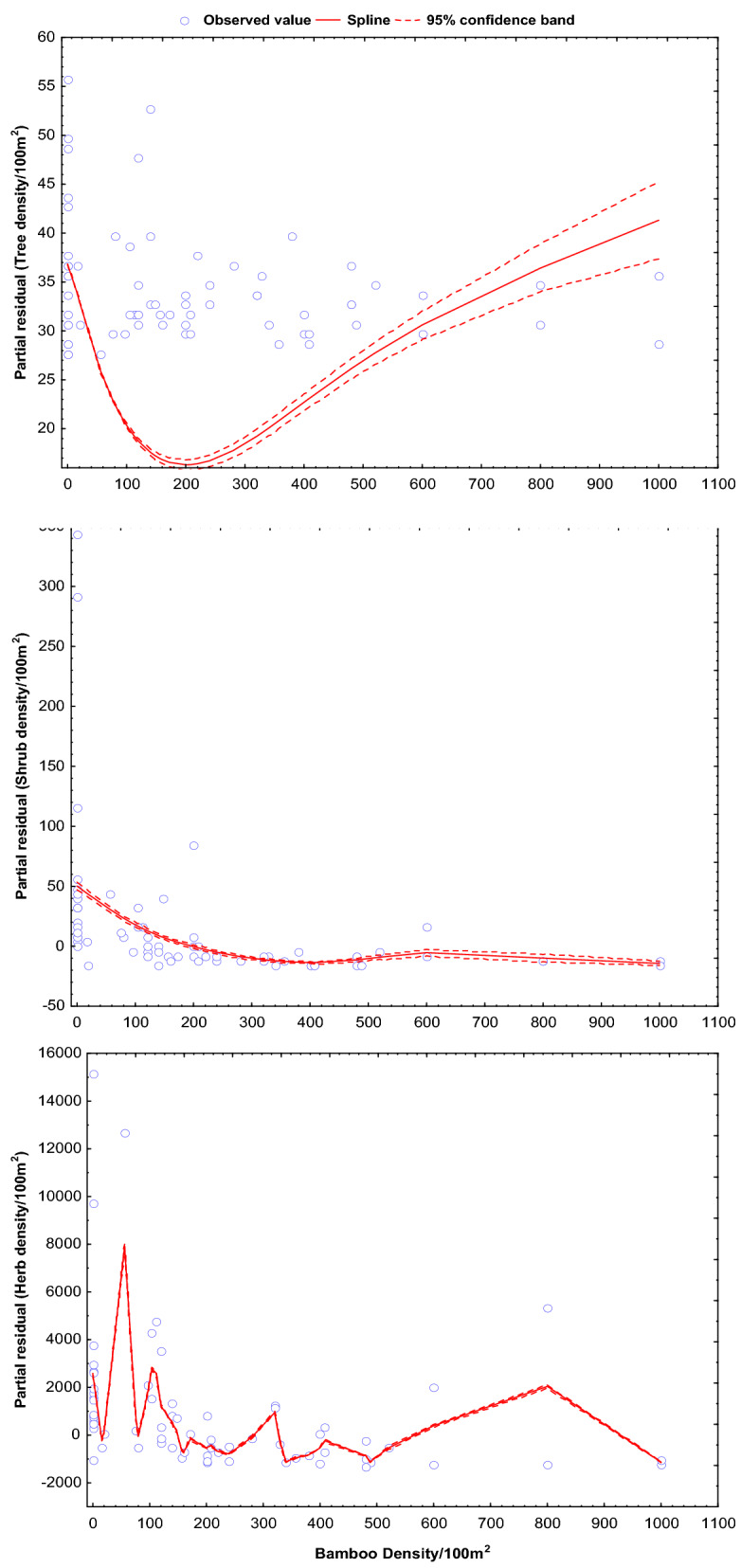
Predicted changes in density of trees, shrubs and herbs with respect to increasing density of bamboo. The significance of the smooth best fit model generalized the changing slope as GAM coefficient (β), standard error (SE) and coefficient of determinant (R^2^).

Concerning the bamboo shoot density across the plots, we estimated the changes in species richness for trees, shrubs, and herbs (Table 4). Our results projected a significantly (p < 0.0001) declining trends for shrubs (β = − 0.0026; SE = 0.0005; R^2^ = 53.61) and a non-significant decline for trees and herbs (Fig. 1) with increasing bamboo density. Similarly, the density indicated declining trends significantly (p < 0.001) for shrubs (β = − 0.029; SE = 0.0013; R^2^ = 43.94) and herbs (β = − 1.956; SE = 0.013; R^2^ = 57.03) (Fig. 2). The model predicted a significant decrease in the richness of shrubs (2–3 species) with an increase of 1000 shoots of bamboo per 100 m^2^ area (Table 4). Also, the model projected significant decline in species density with increasing 1000 shoots of bamboo for shrubs (28–30 individual) and herbs (1943–1969 individual) per 100m^2^ area. Nevertheless, we did not observe the trends for a change in the species richness of trees and herbs, and density of trees (p > 0.05), which indicates recent overdominance of *Y. maling* in the area.

**Table 4:** Estimated changes in species richness and density of trees, shrubs and herbs with respect to bamboo density in the Singalila National Park.

Parameters	GAM coefficient (β)	SE (β)	R^2^	*P*_*value*_
**Species richness**				
Trees	− 0.00004	0.0007	14.29	0.766
Shrubs	− 0.0026	0.0005	53.61	0.0001
Herbs	− 0.0021	0.00002	ne	1.000
**Density**				
Trees	− 0.166	0.0004	6.90	0.844
Shrubs	− 0.029	0.0013	43.94	0.001
Herbs	− 1.956	0.013	57.03	0.001

*ne* Non eligible (as the R^2^ value is < 1).

**Figure 3:**
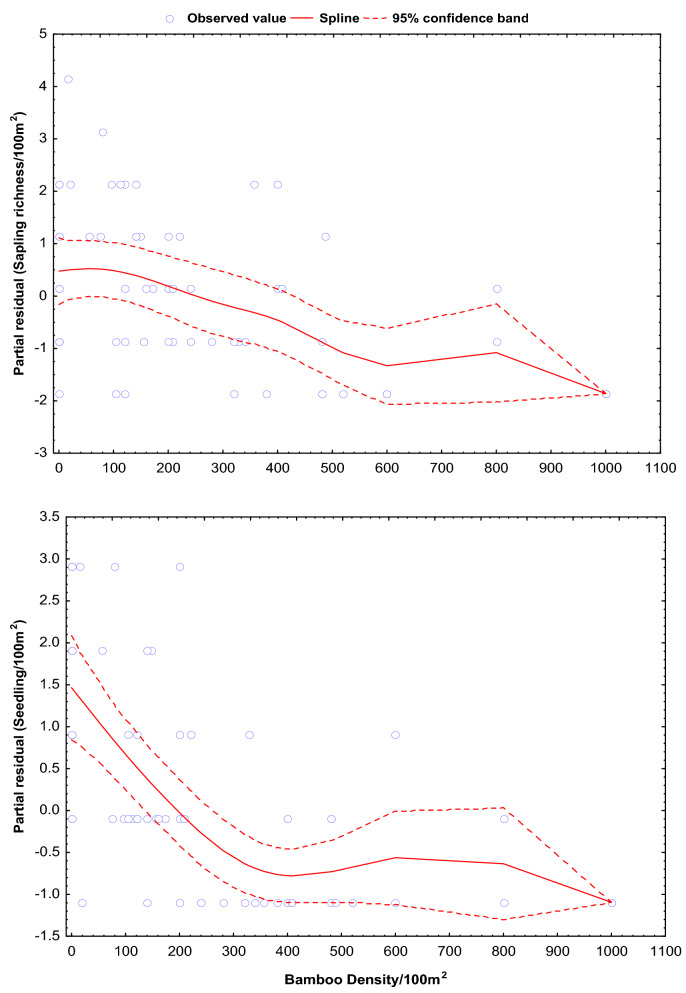
Predicted changes in species richness of tree sapling and seedlings with respect to increasing density of bamboo. The significance of the smooth best fit model generalized the changing slope as GAM coefficient (β), standard error (SE) and coefficient of determinant (R^2^).

**Figure 4:**
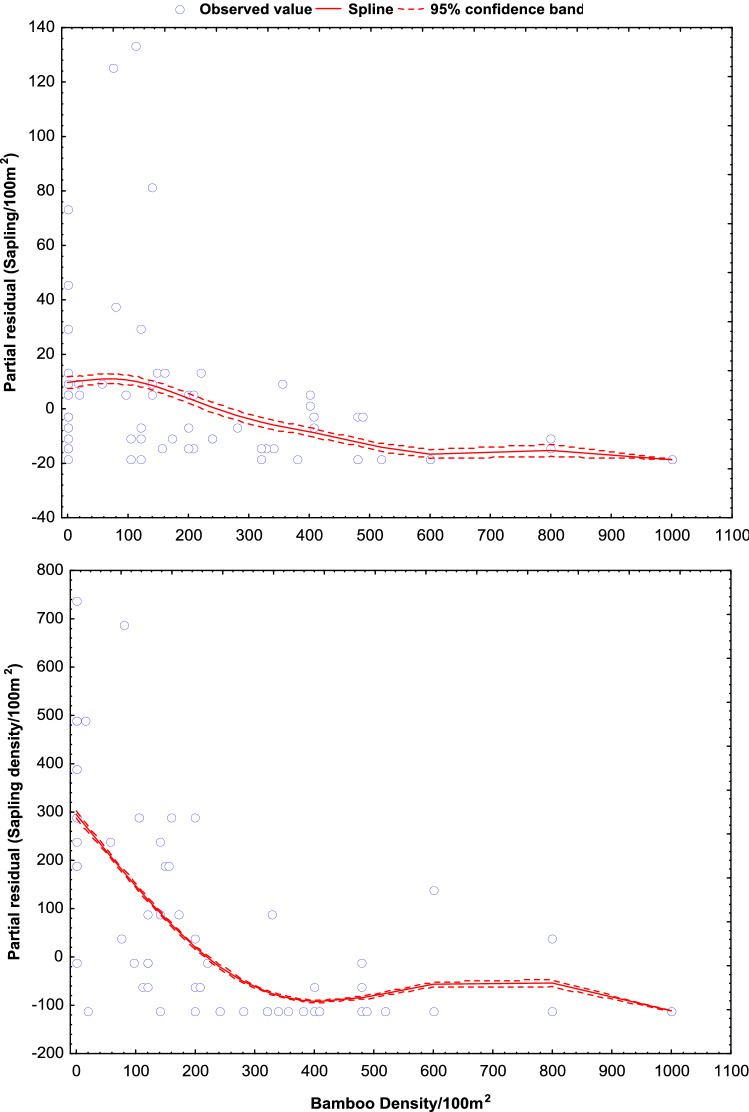
Predicted changes in density of tree sapling and seedlings with respect to increasing density of bamboo. The significance of the smooth best fit model generalized the changing slope as GAM coefficient (β), standard error (SE), coefficient of determinant (R^2^).

The GAMs projected the species richness and density of tree saplings and seedlings along with the bamboo (shoot) density (Table 5). GAM showed a non-significant decline in the species richness of tree saplings (β = − 0.0024; SE = 0.0002; R^2^ = 22.70) and a significant (p < 0.00001) decline seedlings (β = − 0.0014; SE = 0.0002; R^2^ = 45.57) (Fig. 3). Similarly, the density of tree saplings (β = − 0.024; SE = 0.0007; R^2^ = 23.41) and seedlings (β = − 0.143; SE = 0.002; R^2^ = 55.92) were observed decreasing significantly (p < 0.0001) with increasing density of bamboo (shoots) (Fig. 4). Our model predicted a decrease in tree seedlings (1–2 species) richness with an increase of 1000 shoots of bamboo per 100 m^2^. Similarly, tree saplings (23–25 individual) and seedlings (141–145 individual) density were projected as decrease with an increase of 1000 shoots of bamboo per 100 m^2^.

**Table 5:** Estimated changes in species richness and density of saplings and seedlings with respect to bamboo density in the Singalila National Park.

Parameters	GAM-coefficient (β)	SE (β)	R^2^	*P*_value_
**Species richness**				
Saplings	− 0.0024	0.0002	22.70	0.3630
Seedlings	− 0.0014	0.0002	45.57	0.00001
**Density**				
Saplings	− 0.024	0.0007	23.41	0.0001
Seedlings	− 0.143	0.002	55.92	0.0001

## Discussion

In our study in SNP, the plots invaded by bamboo (*Y. maling*) showed the low species richness and density of the shrubs, herbs, and tree seedlings as compared to the non-bamboo plots. These results indicate that the abundance of bamboo restricts the richness and density of the shrubs and herbs species and hinders tree regeneration in the forests. A supportive study on the expansion of dwarf bamboo in Japan^9^ showed the negative relationship between bamboo density and herb species richness. Also, studies have suggested altering forest structure and dynamics, revealing lower density of trees and lower species diversity, by the overabundance of bamboo in Brazilian Atlantic forest^15^. Such evidences signifies that the high degree of bamboo dominance over a long period may effectively alter the forest composition and structures.

Our analysis of the effect of the bamboo overdominance on the forest composition in SNP and the results drawn from GAM vividly estimated the significant decline in the richness of shrubs and herbs and further predicted that, with increasing 1000 shoots of bamboo per 100 m^2^, there would be an adverse change in the species richness of shrubs (2–3 species). Also, changes in the richness of tree seedlings, (1–2 species) were estimated with 1000 shoots of bamboo. Similarly, disadvantageous changes of density of shrubs (28–30 individual) and herbs (1943–1969 individual), tree saplings (23–25 individual) and tree seedlings (141–145 individual) were estimated with increasing the 1000 shoots of bamboo in 100 m^2^ area significantly. The GAMs predictions indicate that the abundance of bamboo has negatively influenced the richness of shrubs, herbs and tree saplings and tree seedlings. Concurrently, the density of shrubs, herbs, tree saplings and tree seedlings in SNP, as the physical and physiological stresses of bamboo, may cause the reduction of the tree seedlings’ richness^15^. Further, it is suggested that the dominance of bamboo reduces the tree regeneration and the undergrowth vegetation, and their thick layer of litter affects the tree seedling regeneration negatively causing alternations in the plant community composition and species diversity^39^. Another supportive study from Southwest China suggested that the high density of bamboo *Fargesia* *nitida* greatly decreases understory species richness especially shrubs^40^. However, the expansion of a dominant native species may threaten other native forest species and enables invasion of non-native species in herbs and shrubs layers^7^.

The rapid overdominance of bamboo species (*Y. maling*) in SNP has consequently indicated the alteration in forest composition when compared with non-bamboo plots. Here, we recorded the widespread extension of bamboo reaching upto 3300 m asl, which is commonly observed in the Temperate broadleaved forests, and expanded across the Temperate Coniferous broad-leaved forests. We observed excellent bamboo growth under tree species like *Quercus* spp*., Rhododendron* spp*., Eurya* spp*., Symploccus* spp*., Vitex heterophyllum* and *Abies densa* accompanying various shrub species because of its tremendous adaptability and excellent resource use (light) capability in different habitats^41^. They expand through their rhizomes and survive over several decades in many forest types and thus have raised concerns about the regeneration of tree seedlings and the diversity of shrubberies and herbaceous species^13,42–44^. Consequently, some studies have suggested controlling and managing the invasion of bamboos by applying moderate grazing. That can control forest structures to some extent thereby reducing the density and height of the bamboos and creating favorable conditions for the regeneration of other species^42,45,46^.

Additionally, our results reveal a positive alteration in species richness of shrubs, herbs, and tree seedlings and in density of tree, shrubs, herbs and tree seedlings while, negative change in tree saplings richness and density of tree saplings and bamboo (stems). Previous study from similar region resulted negative correlation between species richness of woody species and elevation^47^ however, contradictory results as adverse change in species richness along the elevation emerged from the mountains of China^48^. The change in species richness and density may be due to difference in climatic, physiographic and edaphic factors ^2,49^.

In general, bamboos offer major ecosystem services, providing livelihood opportunities and supplying food resources to humans and wild fauna. These uses are wide-ranging, like tender shoots eaten as vegetable, the leaves for fodder to cattle and as important food to the wild animals especially for the endangered animal Red panda (*Ailurus fulgens*)^21,22^ and the Himalayan black Bear (*Selenarctos thibetanus*) in SNP. The bamboo seeds and young shoots are the good food resources for wild herbivores^50,51^. Also, the species offers various uses in constructive purposes, linking directly to the livelihood of the forest fringe communities. Simultaneously, bamboo may provide opportunities for carbon farming and carbon trading^16,52^ and supports the increasing organic matters in soil^53^. Nonetheless, as per the National Park rule and regulations, the community people around SNP are restricted to use bamboo and other resources for the commercial purposes. The invasiveness of native bamboo species (*Y. maling*) in SNP might have resulted, due to, i) ban on grazing and shifting shepherd communities outside the park area, ii) restriction on harvesting bio-resources for the forest fringe communities, iii) global climate change, and iv) limited management measures, etc.

The management of bamboos by controlling their expansion in the areas of high bamboo density could be an appropriate forest restoration approach. It is understood that the bamboos have great potential for the rapid growth and forming complex dominant structures in the forests. The sustainable utilization of bamboos by the local communities would be suggestive especially in the PAs, not only in Darjeeling Himalaya but also in the entire eastern Himalayan forests. Such uses of bamboos can be encouraged, in order to, (i) limit its uncontrolled extension further inside the forests, (ii) decrease extraction pressure in other forest wood species, (iii) revitalize the local bamboo-based traditional handicrafts and linking with livelihood alternatives and (iv) remove bamboo clumps form affected areas, which can be used as a planting material in waste land restoration purpose. Besides, the growing ecotourism in the region demands the supply of various ethnic products for which the bamboo resources become the best option as an alternative livelihood. The bamboo should be used in such a way that the negative impacts of its current expansion on the other species could be limited.

Based on the information gathered from our case study from Khangchendzonga Landscape-India, appropriate management plans need to be developed, focusing on its sustainable harvesting system and identifying the threshold levels of extraction for conservation and sustainable utilization approaches. Furthermore, we suggest in-depth further studies on, (i) bamboo-based forest community structures and dynamics, (ii) changing the intensity of bamboos community growth with respect to climate change, (iii) monitoring ecological resilience with respect to the bamboos plantation and cultivation, and (iv) integrating mechanism of sustainable utilization of bamboo by the communities around PAs.

## Methods

### Study site

**Figure 5:**
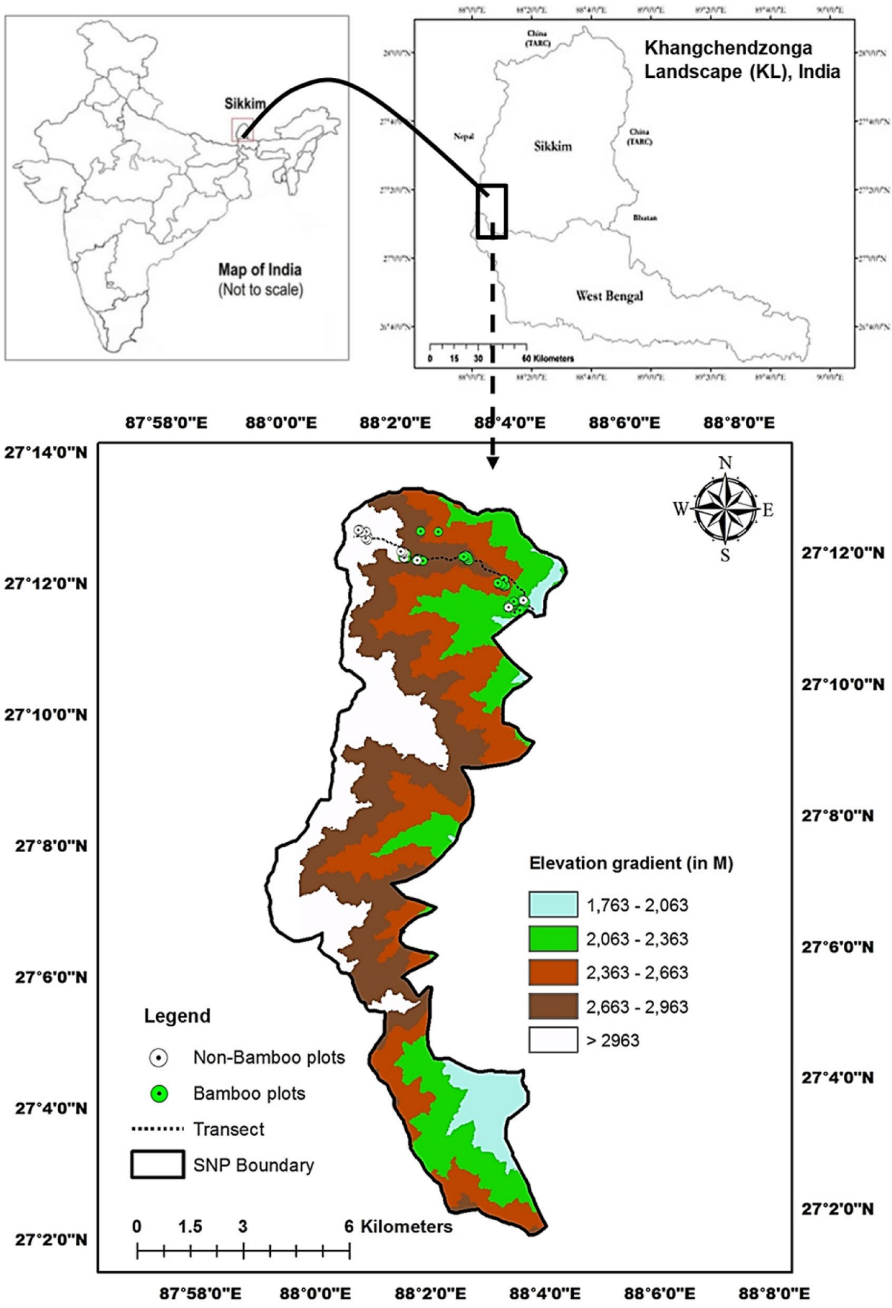
Study transect and location map of Singalila National Park under Khagchendzonga Landscape–India. The map was generated using the software Arc GIS version 9.3.

The Khangchendzonga Landscape (KL) [26° 21′ 40.49″ and 28° 7′ 51.25″ E latitude and 87° 30′ 30.67″ and 90° 24′ 31.18″ N longitude], delineated in January 2014, extends from the southern stretch of Mount Khangchendzonga (8,586 m asl) and spreads over varied ecological zones in eastern Nepal, Sikkim and northern West Bengal in India and western Bhutan. The Landscape covers the significant region of Eastern Himalaya from 40 m asl upto 8,586 m asl^54^. Covering spatial area of 14,126.36 km^2^, the Indian part of KL includes 17 protected areas (PAs), of which four PAs have transboundary settings with neighbouring countries. Our study site, Singalila National Park (SNP), covers an area of 79 km^2^, extending within 27° 13′ 15″ N and 22° 1′ 46″ N latitude and 83° 1′ 91″ to 38° 7′ 54″ E longitude. SNP lies in transboundary with Nepal and India, and its northern part borders with Barsey Rhododendron Sanctuary of Sikkim. The study site represents sub-tropical to sub-alpine eco-climatic zones (2200 m to 3668 m asl) along the trekking corridor from the Gorkhey forest village to Phalut (Fig. 5). The diversified eco-climatic ranges provide various broad habitats and numerous microhabitats harboring the inclusive floral and faunal diversity. A prior permission to conduct this study was taken from the Department of Forests, West Bengal and all rules and regulations of protected areas (National Park) were followed during the study.

### Sampling design

The study was undertaken along the stretch of 2400 m asl to 3400 m asl (temperate to sub-alpine zone) in SNP, across the Gorkhey-Phalut trekking corridor. In the region below 2400 m, the bamboo growth was sparse due to the anthropogenic activity including the extent of agricultural fields and the use of bamboo for various purposes by the people of the fringe villages. Along this elevation, we randomly sampled 69 plots to assess the community structure of the vegetation (tree, shrub and herbaceous species) within the assemblage of *Y. maling*, as the major understory growth including both bamboo and non-bamboo areas^55^. We adopted nested quadrat method for the study of species richness and density of trees, shrubs, herbs, tree saplings and tree seedlings within identified plots. We studied the tree species in 0.1 hectare (10 m ×10 m) plots, within which 25 m^2^ (5 m × 5 m) plots exactly in the middle were selected to study shrubs, bamboos, and tree saplings. We laid two quadrats m^2^ (1 m × 1 m) each in the alternative corners of the major plot to study tree seedlings and other herbaceous species. We considered mature trees having the girth size ≥ 30 cm (girth at breast height; 1.3 cm above the ground level); the saplings having girth of < 30 cm to ≥ 10 cm, while we regarded seedlings having collar girth < 10 cm /or collar diameter < 3.14 cm at the height of 1 cm from the ground level. In all the plots, we recorded total number and the respective diameters of each tree. Measuring tape was used to measure the tree/ sapling circumference, however, seedling diameter was measured with the help of digital Vernier caliper (Model:CD-8 ˝CS, Mituyoto, Japan). However, Small-to-medium sized woody perennial plants having profuse branching from the base were considered shrub. However, most annuals and few perennials with herbaceous stem were kept under herb layer. To understand the bamboo density responses over other forest communities (trees, shrubs, herbs, including seedling and sapling of trees), we recorded it in each random plot. Out of 69 major 0.1 ha plots, 51 were recognized with the occurrence of bamboo species and 18 plots without any presence of *Y. maling*. Plant species of each plot were identified using filed guide books and their identity was confirmed by consulting regional floras (Flowers of the Himalaya^56^; Flora of Darjeeling Himalayas and Foothills: Angiosperms^57^; Sikkim Himalayan Rhododendrons^58^) and available herbarium records. The plant species nomenclature follows the Plant list (www.theplantlist.org) an online database accessed on 01/01/2022. For the quantitative measure of species richness (tree, shrub, herb, sapling, and seedling) was calculated as the number of species per unit area ^59^. We measured the density (number of individuals per unit area) for tree, shrub, herb, tree sapling, and tree seedling, simultaneously with *Y. maling* density. For bamboo density, shoots/culms were counted from each sampled plot.

### Statistical analysis

Realizing the asymmetrical data structure (Non-Normal), we transformed the recorded data into arcsine format and applied independent sample t-test for comparing the mean of diversity and density of the trees, shrubs, herbs, and seedlings and saplings of trees between bamboo and non-bamboo plots. Finally, we retransformed the analysed arcsine data presented in the tables for interpretation.

To deal with nonparametric statistical analysis, Generalized Additive Model (GAM) was considered exceedingly suitable for ecological studies^60,61^. The GAM is a semi-parametric extension of GLM^62^ and deals with highly non-linear and non-normal relationships between the response and the set of explanatory variables^63^. GAM includes the estimation of smoothing terms in the additive model and general algorithm added in the model as partial residuals (i.e. *R*_*j*_^*th*^ set of partial residuals).

$$R_{ij} = Y - S_{0} - \sum\limits_{h \equiv \ne j} {S_{k} (X_{k} )}$$

The partial residuals remove the effects of all the other variables from *Y* (depended variable i.e. richness and density of trees, shrubs, herbs, and saplings and seedlings of trees); therefore, the *Y* can be used to model the effects against *X*_*j*_ (density of bamboo-*Y. maling* for all studied plots including non-bamboo plots as zero quantitative value). However, due to ranging wider variability and inconsistence series of data, the variables under study were indicated non-normal and non-linear data structure.

## Supplementary Information

Supplementary Information.
