# Supplementary material for: Maling bamboo (Yushania maling) overdominance alters forest structure and composition in Khangchendzonga landscape, Eastern Himalaya
Source: Sci Rep. 2022 Mar 16;12:4468. doi: 10.1038/s41598-022-08483-8 (PMC8927343; doi:10.1038/s41598-022-08483-8)
Supplement: Supplementary file 1 — Supplementary Information. [file 41598_2022_8483_MOESM1_ESM.docx]

**Supplementary table 1: Enumerated plant species check list of Singalila National Park, Eastern Himalaya, India**

| **SN** | **Plant species** | **Local Name** | **Family** |
| --- | --- | --- | --- |
| **Tree layer** | | | |
|  | *Abies* *densa* Griff. | Gobre Salla | Pinaceae |
|  | *Acer* *campbellii* Hook.f. & Thomson ex Hiern | Kapase | [Sapindaceae](http://www.theplantlist.org/1.1/browse/A/Sapindaceae/) |
|  | *Acer* *laevigatum* Wall. | Putli | [Sapindaceae](http://www.theplantlist.org/1.1/browse/A/Sapindaceae/) |
|  | *Aralia* *leschenaultii* (DC.) J.Wen | Chinde | [Araliaceae](http://www.theplantlist.org/1.1/browse/A/Araliaceae/) |
|  | *Betula* *utilis* D.Don | Bhoj Patra | [Betulaceae](http://www.theplantlist.org/1.1/browse/A/Betulaceae/) |
|  | *Castanopsis* *tribuloides* (Sm.) A.DC. | Katus | [Fagaceae](http://www.theplantlist.org/1.1/browse/A/Fagaceae/) |
|  | *Edgeworthia* *gardneri* (Wall.) Meisn. | Argeli | [Thymelaeaceae](http://www.theplantlist.org/1.1/browse/A/Thymelaeaceae/) |
|  | *Eurya* *acuminata* DC. | Jhigani | [Pentaphylacaceae](http://www.theplantlist.org/1.1/browse/A/Pentaphylacaceae/) |
|  | *Ilex* *sikkimensis* Kurz | Sikkim Holly | [Aquifoliaceae](http://www.theplantlist.org/1.1/browse/A/Aquifoliaceae/) |
|  | *Lithocarpus* *pachyphyllus* (Kurz) Rehder | Sungurae katus, Bantey katus | Fagaceae |
|  | *Litsea* *cubeba* (Lour.) Pers. | Sil Timbur | [Lauraceae](http://www.theplantlist.org/1.1/browse/A/Lauraceae/) |
|  | *Litsea* *elongata* (Nees) Hook. f. | Pahele | [Lauraceae](http://www.theplantlist.org/1.1/browse/A/Lauraceae/) |
|  | *Machilus* *edulis* King ex Hook.f. | Kawlo | Lauraceae |
|  | *Magnolia* *doltsopa* (Buch.-Ham. ex DC.) Figlar | Rani Chanp | [Magnoliaceae](http://www.theplantlist.org/1.1/browse/A/Magnoliaceae/) |
|  | *Meliosma* *arnottiana* (Wight) Walp. | Dabdabbi | [Sabiaceae](http://www.theplantlist.org/1.1/browse/A/Sabiaceae/) |
|  | *Osmanthus* *suavis* King ex C.B.Clarke | Seti Kath | [Oleaceae](http://www.theplantlist.org/1.1/browse/A/Oleaceae/) |
|  | *Pieris* *formosa* (Wall.) D. Don | Ballu | [Ericaceae](http://www.theplantlist.org/1.1/browse/A/Ericaceae/) |
|  | *Prunus* *bracteopadus* Koehne | Arupatey | [Rosaceae](http://www.theplantlist.org/1.1/browse/A/Rosaceae/) |
|  | *Prunus* *undulata* Buch.-Ham. ex D.Don | Arupatey | [Rosaceae](http://www.theplantlist.org/1.1/browse/A/Rosaceae/) |
|  | *Quercus* *glauca* Thunb. | Sanu Phalat | [Fagaceae](http://www.theplantlist.org/1.1/browse/A/Fagaceae/) |
|  | *Quercus* *lamellosa* Sm. | Buk, Bajrath | [Fagaceae](http://www.theplantlist.org/1.1/browse/A/Fagaceae/) |
|  | *Quercus* *lineata* Blume | Phalat | [Fagaceae](http://www.theplantlist.org/1.1/browse/A/Fagaceae/) |
|  | *Quercus* *thomsoniana* A.DC. | Katus | [Fagaceae](http://www.theplantlist.org/1.1/browse/A/Fagaceae/) |
|  | *Rhododendron* *arboreum* Sm. | Laligurans | [Ericaceae](http://www.theplantlist.org/1.1/browse/A/Ericaceae/) |
|  | *Rhododendron* *barbatum* Wall. ex G. Don | Junge Chimal | [Ericaceae](http://www.theplantlist.org/1.1/browse/A/Ericaceae/) |
|  | *Rhododendron* *falconeri* Hook.f. | Korlinga | Ericaceae |
|  | *Rhododendron* *fulgens* Hook. f. | Pahelo Korlingo | [Ericaceae](http://www.theplantlist.org/1.1/browse/A/Ericaceae/) |
|  | *Rhododendron* *grande* Wight | Chimal | [Ericaceae](http://www.theplantlist.org/1.1/browse/A/Ericaceae/) |
|  | *Schefflera* *rhododendrifolia* (Griff.) Frodin | Bhalu Chinde | [Araliaceae](http://www.theplantlist.org/1.1/browse/A/Araliaceae/) |
|  | *Symplocos* *glomerata* King ex C.B. Clarke | Kholmae | [Symplocaceae](http://www.theplantlist.org/1.1/browse/A/Symplocaceae/) |
|  | *Symplocos* *lucida* (Thunb.) Siebold & Zucc. | Kharane | [Symplocaceae](http://www.theplantlist.org/1.1/browse/A/Symplocaceae/) |
|  | *Taxus* *baccata* L. | Thingrae Salla | [Taxaceae](http://www.theplantlist.org/1.1/browse/G/Taxaceae/) |
|  | *Thuja* *occidentalis* L. | Dhupi | [Cupressaceae](http://www.theplantlist.org/1.1/browse/G/Cupressaceae/) |
|  | *Tsuga* *dumosa* (D.Don) Eichler | Dhengre Salla | [Pinaceae](http://www.theplantlist.org/1.1/browse/G/Pinaceae/) |
|  | *Viburnum* *nervosum* D. Don | Asare | [Adoxaceae](http://www.theplantlist.org/1.1/browse/A/Adoxaceae/) |
|  | *Zanthoxylum* *acanthopodium* DC. | Bokey Timbur | [Rutaceae](http://www.theplantlist.org/1.1/browse/A/Rutaceae/) |
| **Shrub and woody climber layer** | | | |
| 1. | *Actinidia* *callosa* Lindl. | Theki Fal | [Actinidiaceae](http://www.theplantlist.org/1.1/browse/A/Actinidiaceae/) |
| 2. | *Agapetes* *serpens* (Wight) Sleumer | Khorsani | [Ericaceae](http://www.theplantlist.org/1.1/browse/A/Ericaceae/) |
| 3. | *Berberis* *aristata* DC. | Chutre | [Berberidaceae](http://www.theplantlist.org/1.1/browse/A/Berberidaceae/) |
| 4. | *Brugmansia suaveolens (Humb. & Bonpl. ex Willd.) Bercht. & J.Presl* | Dhokreyphul | [Solanaceae](http://www.theplantlist.org/1.1/browse/A/Solanaceae/) |
| 5. | *Daphne* *papyracea* Wall. ex G. Don | Lokti | [Thymelaeaceae](http://www.theplantlist.org/1.1/browse/A/Thymelaeaceae/) |
| 6. | *Daphne* *sureil* W.W.Sm. & Cave | Lokti | [Thymelaeaceae](http://www.theplantlist.org/1.1/browse/A/Thymelaeaceae/) |
| 7. | *Elaeagnus* *latifolia* L. | Musledi | [Elaeagnaceae](http://www.theplantlist.org/1.1/browse/A/Elaeagnaceae/) |
| 8. | *Gaultheria* *nummularioides* D.Don | Dhasingre | [Ericaceae](http://www.theplantlist.org/1.1/browse/A/Ericaceae/) |
| 9. | *Gaultheria* *pyroloides* Hook.f. & Thomson ex Miq. | Dhasingre | [Ericaceae](http://www.theplantlist.org/1.1/browse/A/Ericaceae/) |
| 10 | *Gaultheria* *trichophylla* Royle | Kali Gedi | [Ericaceae](http://www.theplantlist.org/1.1/browse/A/Ericaceae/) |
| 11. | *Hypericum* *elodeoides* Choisy | Sirilnge | [Hypericaceae](http://www.theplantlist.org/1.1/browse/A/Hypericaceae/) |
| 12. | *Osbeckia* *stellata* Buch.-Ham. ex Ker Gawl. | Lote | [Melastomataceae](http://www.theplantlist.org/1.1/browse/A/Melastomataceae/) |
| 13. | *Persicaria* *wallichii* Greuter & Burdet | Rani Thotne | [Polygonaceae](http://www.theplantlist.org/1.1/browse/A/Polygonaceae/) |
| 14. | *Phlogacanthus* *thyrsiflorus* Nees | Titae | Acanthaceae |
| 15. | *Piper* *hamiltonii* C.DC. | Pan Pate | Piperaceae |
| 16. | *Polygonum* *molle* D. Don | Thotne | [Polygonaceae](http://www.theplantlist.org/1.1/browse/A/Polygonaceae/) |
| 17. | *Rhododendron lepidotum Wall. ex G. Don* | Sunpatey | [Ericaceae](http://www.theplantlist.org/1.1/browse/A/Ericaceae/) |
| 18. | *Rosa sericea Wall. ex Lindl.* | Jungali Gulab | [Rosaceae](http://www.theplantlist.org/1.1/browse/A/Rosaceae/) |
| 19. | *Rubia* *manjith* Roxb. ex Fleming | Majhito | [Rubiaceae](http://www.theplantlist.org/1.1/browse/A/Rubiaceae/) |
| 20. | *Rubus* *ellipticus* Sm. | Aiselu | [Rosaceae](http://www.theplantlist.org/1.1/browse/A/Rosaceae/) |
| 21. | *Rubus lineatus Reinw. ex Blume* | Putali Kara | [Rosaceae](http://www.theplantlist.org/1.1/browse/A/Rosaceae/) |
| 22. | *Rubus nepalensis hort.* | Bhui Aiselu | [Rosaceae](http://www.theplantlist.org/1.1/browse/A/Rosaceae/) |
| 23. | *Schisandra* *neglecta* A.C.Sm. | Singoto | [Schisandraceae](http://www.theplantlist.org/1.1/browse/A/Schisandraceae/) |
| 24. | *Sorbus* *rhamnoides* (Decne.) Rehder | Pasi Lahara | [Rosaceae](http://www.theplantlist.org/1.1/browse/A/Rosaceae/) |
| 25. | *Stephania glabra (Roxb.)* Miers | Tamarkey | Menispermaceae |
| **Bamboos** | | | |
| 1. | *Drepanostachyum* *falcatum* (Nees) Keng f. | Nigalo | [Poaceae](http://www.theplantlist.org/1.1/browse/A/Poaceae/) |
| 2. | *Yushania* *maling* (Gamble) R.B.Majumdar & Karthik. | Malling | [Poaceae](http://www.theplantlist.org/1.1/browse/A/Poaceae/) |
| **Herb layer** | | | |
| 1. | *Aconitum* *ferox* Wall. ex Ser. | Bikhma | [Ranunculaceae](http://www.theplantlist.org/1.1/browse/A/Ranunculaceae/) |
| 2. | *Ageratina* *adenophora* (Spreng.) R.M.King & H.Rob. | Kali Jhar | [Compositae](http://www.theplantlist.org/1.1/browse/A/Compositae/) |
| 3. | *Anaphalis* *contorta* (D.Don) Hook.f. | Bukey Phool | [Compositae](http://www.theplantlist.org/1.1/browse/A/Compositae/) |
| 4. | *Anaphalis* *margaritaceae* L. | Bukey Phool | [Compositae](http://www.theplantlist.org/1.1/browse/A/Compositae/) |
| 5. | *Cardamine* *macrophylla* Willd. | Bhotey Saag | [Brassicaceae](http://www.theplantlist.org/1.1/browse/A/Brassicaceae/) |
| 6. | *Carex* *baccans* Nees | Ban Kodo | [Cyperaceae](http://www.theplantlist.org/1.1/browse/A/Cyperaceae/) |
| 7. | *Carex* *inanis* Kunth | Harkatta | [Cyperaceae](http://www.theplantlist.org/1.1/browse/A/Cyperaceae/) |
| 8. | *Cautleya* *spicata* (Sm.) Baker | Ban Hardi | [Zingiberaceae](http://www.theplantlist.org/1.1/browse/A/Zingiberaceae/) |
| 9. | *Cirsium* *verutum* (D.Don) Spreng. | Thakal | [Compositae](http://www.theplantlist.org/1.1/browse/A/Compositae/) |
| 10. | *Cissus* *elongata* Roxb. | Charchare | [Vitaceae](http://www.theplantlist.org/1.1/browse/A/Vitaceae/) |
| 11. | *Cotula* *hemisphaerica* Wall. ex Benth. & Hook.f. | Gandhe Jhar | [Compositae](http://www.theplantlist.org/1.1/browse/A/Compositae/) |
| 12. | *Curcuma* sp. | Hardi | [Zingiberaceae](http://www.theplantlist.org/1.1/browse/A/Zingiberaceae/) |
| 13. | *Drymaria* *cordata* (L.) Willd. ex Schult. | Abijalo | [Caryophyllaceae](http://www.theplantlist.org/1.1/browse/A/Caryophyllaceae/) |
| 14. | *Elatostema* *platyphyllum* Wedd. | Gogleto | [Urticaceae](http://www.theplantlist.org/1.1/browse/A/Urticaceae/) |
| 15. | *Elatostema* *sessile* J.R.Forst. & G.Forst. | Sim Gogleto | [Urticaceae](http://www.theplantlist.org/1.1/browse/A/Urticaceae/) |
| 16. | *Elsholtzia* *fruticosa* (D.Don) Rehder | Bhote Pati | [Lamiaceae](http://www.theplantlist.org/1.1/browse/A/Lamiaceae/) |
| 17. | *Emilia* *sonchifolia* (L.) DC. ex DC. | Mula Pate | [Compositae](http://www.theplantlist.org/1.1/browse/A/Compositae/) |
| 18. | *Fragaria* *daltoniana* J.Gay | Bhui Aiselu | [Rosaceae](http://www.theplantlist.org/1.1/browse/A/Rosaceae/) |
| 19. | *Fragaria* *nubicola* (Lindl. ex Hook.f.) Lacaita | Bhui Aiselu | [Rosaceae](http://www.theplantlist.org/1.1/browse/A/Rosaceae/) |
| 20. | *Girardinia* *palmata* (Forssk.) Gaudich. | Bhangre | [Urticaceae](http://www.theplantlist.org/1.1/browse/A/Urticaceae/) |
| 21. | *Hedychium* sp. | Saro | [Zingiberaceae](http://www.theplantlist.org/1.1/browse/A/Zingiberaceae/) |
| 22. | *Hemarthria* *compressa* (L.f.) R.Br. | Ghode Dubo | [Poaceae](http://www.theplantlist.org/1.1/browse/A/Poaceae/) |
| 23. | *Hemiphragma* *heterophyllum* Wall. | Lal Geri | [Plantaginaceae](http://www.theplantlist.org/1.1/browse/A/Plantaginaceae/) |
| 24. | *Heracleum* *candicans* Wall. ex DC. | Chimfing | [Apiaceae](http://www.theplantlist.org/1.1/browse/A/Apiaceae/) |
| 25. | *Herpetospermum* *darjeelingense* (C.B.Clarke) H.Schaef. & S.S.Renner | Ban Karela | [Cucurbitaceae](http://www.theplantlist.org/1.1/browse/A/Cucurbitaceae/) |
| 26. | *Impatiens* *kingii* Hook.f. | Tiuri Phool | [Balsaminaceae](http://www.theplantlist.org/1.1/browse/A/Balsaminaceae/) |
| 27. | *Impatiens* *lutea* Lam. | Tiuri Phool | [Balsaminaceae](http://www.theplantlist.org/1.1/browse/A/Balsaminaceae/) |
| 28. | *Imperata* *cylindrica* (L.) Raeusch. | Siru | [Poaceae](http://www.theplantlist.org/1.1/browse/A/Poaceae/) |
| 29. | *Iris* *decora* Wall. | Lekh Bojo | [Iridaceae](http://www.theplantlist.org/1.1/browse/A/Iridaceae/) |
| 30. | *Mimosa* *pudica* L. | Bhowari | [Leguminosae](http://www.theplantlist.org/1.1/browse/A/Leguminosae/) |
| 31. | *Nicotiana* Sp. | Kancho Pat | [Solanaceae](http://www.theplantlist.org/1.1/browse/A/Solanaceae/) |
| 32. | *Oplismenus* *burmanni* (Retz.) P.Beauv. | Banso | [Poaceae](http://www.theplantlist.org/1.1/browse/A/Poaceae/) |
| 33. | *Oxalis* *corniculata* L. | Chari Amilo | [Oxalidaceae](http://www.theplantlist.org/1.1/browse/A/Oxalidaceae/) |
| 34. | *Paris* *polyphylla* Sm. | Satua | [Melanthiaceae](http://www.theplantlist.org/1.1/browse/A/Melanthiaceae/) |
| 35. | *Persicaria* *capitata* (Buch.-Ham. ex D.Don) H.Gross | Ratnaulo | [Polygonaceae](http://www.theplantlist.org/1.1/browse/A/Polygonaceae/) |
| 36. | *Phytolacca* *acinosa* Roxb. | Jaringo | [Phytolaccaceae](http://www.theplantlist.org/1.1/browse/A/Phytolaccaceae/) |
| 37. | *Pilea* *elliptica* Wedd. | Chipley | [Urticaceae](http://www.theplantlist.org/1.1/browse/A/Urticaceae/) |
| 38. | *Plantago* *major* L. | Sime | [Plantaginaceae](http://www.theplantlist.org/1.1/browse/A/Plantaginaceae/) |
| 39. | *Rumex* *nepalensis* Spreng. | Halhalley | [Polygonaceae](http://www.theplantlist.org/1.1/browse/A/Polygonaceae/) |
| 40. | *Senecio* *scandens* Buch.-Ham. ex D.Don | Pahaley | [Compositae](http://www.theplantlist.org/1.1/browse/A/Compositae/) |
| 41. | *Setaria* *palmifolia* (J.Koenig) Stapf | Nariyal Ghas, Dhotisaro | [Poaceae](http://www.theplantlist.org/1.1/browse/A/Poaceae/) |
| 42. | *Smilax* *elegans* Wall. ex Kunth | Kukkur Dane | [Smilacaceae](http://www.theplantlist.org/1.1/browse/A/Smilacaceae/) |
| 43. | *Strobilanthes* *capitata* (Nees) T. Anderson | Akhlae | [Acanthaceae](http://www.theplantlist.org/1.1/browse/A/Acanthaceae/) |
| 44. | *Swertia* *bimaculata* (Siebold & Zucc.) Hook. f. & Thomson ex C.B. Clarke | Bhale Chiraito | [Gentianaceae](http://www.theplantlist.org/1.1/browse/A/Gentianaceae/) |
| 45. | *Swertia* *chirayta* H.Karst. | Chiraito | [Gentianaceae](http://www.theplantlist.org/1.1/browse/A/Gentianaceae/) |
| 46. | *Viola* *pilosa* Blume | Bhush Pate | [Violaceae](http://www.theplantlist.org/1.1/browse/A/Violaceae/) |
